# Supplementary material for: A Practical Guide to Surface Kinetic Monte Carlo Simulations
Source: Front Chem. 2019 Apr 9;7:202. doi: 10.3389/fchem.2019.00202 (PMC6465329; doi:10.3389/fchem.2019.00202)
Supplement: Supplementary file 1 [file Data_Sheet_1.ZIP › SI_data/~WRL3935.tmp]

Additional explanations to the KMOS Python scripts provided as supporting information.

The Python scripts named “render_XXX.py” define the KMC models discussed in the main text. Here XXX refers to:

Au100_diffusion: Adatom diffusion on Au(100) model discussed in Section 5.1

COoxRuO2 and COoxRuO2_lat_int: CO oxidation on RuO_2_(110) model with and without lateral interactions discussed in Section 6.1 and 9.1

SOSadsdes: Solid-on-solid crystal growth model discussed in Section 9.1.

The Python scripts named “run_XXX.py” or “view_XXX.py” are used to run the model, and in the case of the crystal growth model to view the grown structure.
