# Supplementary material for: A Practical Guide to Surface Kinetic Monte Carlo Simulations
Source: Front Chem. 2019 Apr 9;7:202. doi: 10.3389/fchem.2019.00202 (PMC6465329; doi:10.3389/fchem.2019.00202)
Supplement: Supplementary file 1 [file Data_Sheet_1.ZIP › SI_data/Additional_explanations_KMOS_scripts.pdf]

Additional explanations to the KMOS Python scripts

The Python scripts named “render_XXX.py” define the KMC models discussed in the main text. Here XXX refers to:
Au100_diffusion: Adatom diffusion on Au(100) model discussed in Section 5.1
COoxRuO2 and COoxRuO2_lat_int: CO oxidation on RuO_2(110) model with and without lateral interactions discussed in Section 6.1 and 9.1
SOSadsdes: Solid-on-solid crystal growth model discussed in Section 9.1.

For instructions how to install KMOS and its dependencies, more information on how to set up KMOS models and additional explanations of the various input structures and parameters we refer to the KMOS documentation: https://kmos.readthedocs.io/en/latest/.
Further tutorials can be found at: https://github.com/jmlorenzi/intro2kmos

The Python scripts named “run_XXX.py” are used to run the model and to plot selected output using the Python plotting library matplotlib (http://matplotlib.org). For the crystal growth model the “view_SOSadsdes.py” script is used to visualize the grown structure using the Atomic Simulation Environment (ASE) software package (https://wiki.fysik.dtu.dk/ase/).

In the following we suggest some parameters that can be varied in the individual models to get a feeling for how they affect the results.

Au100_diffusion:
In the top of the script various model parameters are defined. Try to vary the parameter “conc” to see how the concentration of particles on the lattice influences the diffusion constant. The parameter “Nruns” defined the number of trajectories to average over. Try to decrease the value and observe how the statistical error on the diffusion constant increases (e.g. calculate the mean and the standard error of the mean). You can also try to play around with the barriers for hopping and exchange diffusion defined a bit further down in the script.

COoxRuO2:
In the top of the script various model parameters are defined. You can for example change how the lattice should be initialized, i.e. which species to add to the cus sites, through the “species” parameter. You can also modify the script further to make your own custom initialization. Finally, you can try to change the temperature as well as the partial pressures of CO and O_2.

SOSadsdes:
In the top of the script various model parameters are defined. Try to change the growth temperature, the rate constant for adsorption, the size of the simulation box as well as the target number of layers to grow.
Hint: Make sure that the grown structure does not extend beyond the size of the simulation box, or you will encounter an error.
You can use the “view_SOSadsdes.py” to visualize the grown structures.
